# Essential of Minor Surgery and Bandaging

**Published:** 1892-07

**Authors:** 


ESSENTIALS OF MINOR SURGERY AND BANDAGING.—With an appendix on venereal diseases. Arranged in the form of questions and answers, prepared especially for students of medicine. By Edward Martin, A. M., M. D., Instructor in Operative Surgery, University of Pennsylvania; Surgeon to the Howard Hospital; Assistant Surgeon to the University Hospital; Author of "Essentials of Surgery," etc., 166

pages, illustrated. Published by W. B. Saunders, Philadelphia.

This work is published as one of "Saunders' Question Compends," series. The subjects treated are well selected, and will prove a valuable aid to the student, in acquiring a concise knowledge of the primary principles essential to a knowledge of the subject treated. W. F. W.

